# Supplementary material for: Seroconversion stages COVID19 into distinct pathophysiological states
Source: eLife. 2021 Mar 16;10:e65508. doi: 10.7554/eLife.65508 (PMC7963480; doi:10.7554/eLife.65508)
Supplement: Transparent reporting form [file elife-65508-transrepform.pdf]

eLIFE
1st Floor
24 Hills Road
Cambridge CB2 1JP, UK
P 01223 855340
W elifesciences.org
T @elife

eLife's transparent reporting form

We encourage authors to provide detailed information *within their submission* to facilitate the interpretation and replication of experiments. Authors can upload supporting documentation to indicate the use of appropriate reporting guidelines for health-related research (see EQUATOR Network), life science research (see the BioSharing Information Resource), or the ARRIVE guidelines for reporting work involving animal research. Where applicable, authors should refer to any relevant reporting standards documents in this form.

If you have any questions, please consult our Journal Policies and/or contact us: editorial@elifesciences.org.

Sample-size estimation

- You should state whether an appropriate sample size was computed when the study was being designed
- You should state the statistical method of sample size computation and any required assumptions
- If no explicit power analysis was used, you should describe how you decided what sample (replicate) size (number) to use

Please outline where this information can be found within the submission (e.g., sections or figure legends), or explain why this information doesn't apply to your submission:

Sample size was estimated based on previous published studies investigating autoinflammatory processes in individuals with Down syndrome using technologies identical to those employed in this study, such as cytokine profiling using multiplexed immunoassays (e.g. Powers et al, Nature Communications 2019; Araya et al, PNAS 2019); SOMAscan® proteomics (e.g. Sullivan et al, Scientific Reports 2017); and RNAseq transcriptome analysis (e.g. Powers et al, Nature Communications 2019; Araya et al, PNAS 2019). A formal power analysis was not performed before deciding on sample size for this study. Instead, based on our studies of interferon-driven inflammation in Down syndrome, and assuming similar or greater size effects in COVID19, we estimated that a sample size of 30+ controls versus 70+ COVID19 patients would suffice to identify statistically significant changes in cytokines, plasma proteins, mRNAs, and metabolites. This information can be found in the Materials and Methods section.

Replicates

- You should report how often each experiment was performed
- You should include a definition of biological versus technical replication
- The data obtained should be provided and sufficient information should be provided to indicate the number of independent biological and/or technical replicates
- If you encountered any outliers, you should describe how these were handled
- Criteria for exclusion/inclusion of data should be clearly stated
- High-throughput sequence data should be uploaded before submission, with a private link for reviewers provided (these are available from both GEO and ArrayExpress)

Please outline where this information can be found within the submission (e.g., sections or figure legends), or explain why this information doesn't apply to your submission:

eLife Sciences Publications, Ltd is a limited liability non-profit non-stock corporation incorporated in the State of Delaware, USA, with company number 5030732, and is registered in the UK with company number FC030576 and branch number BR015634 at the address 1st Floor, 24 Hills Road, Cambridge CB2 1JP | August 2014

Seroconversion assays were performed in duplicate for each plasma sample, multiplexed immunoassays for cytokines were performed in duplicate for each plasma sample, mass cytometry immune mapping was performed once for each individual fraction of PBMCs, SOMAscan® proteomics was completed once for each plasma sample, RNAseq was performed once for each whole blood RNA sample, and mass spectrometry proteomics was performed once for each plasma sample. Each research participant is considered a biological replicate for the purpose of the comparisons in this study, such as COVID19 negative versus COVID19 positive, or sero-low versus sero-high groups among COVID19 patients. Extreme outlier data points (above Q3 + 3xIQR or below Q1 – 3XIQR) were removed. This information can be found in the Materials and Methods section.

Statistical reporting

- Statistical analysis methods should be described and justified
- Raw data should be presented in figures whenever informative to do so (typically when N per group is less than 10)
- For each experiment, you should identify the statistical tests used, exact values of N, definitions of center, methods of multiple test correction, and dispersion and precision measures (e.g., mean, median, SD, SEM, confidence intervals; and, for the major substantive results, a measure of effect size (e.g., Pearson's r, Cohen's d)
- Report exact p-values wherever possible alongside the summary statistics and 95% confidence intervals. These should be reported for all key questions and not only when the p-value is less than 0.05.

Please outline where this information can be found within the submission (e.g., sections or figure legends), or explain why this information doesn't apply to your submission:

The statistical analysis pipeline is described in detail in the Materials and Methods section. Data points are presented in the figures in the form of XY scatter plots and/or Sina plots. Methods of multiple test correction, dispersion, and precision are described in Figure legends. p values and q values are displayed for each graph. Statistical tests employed are described in each Figure legend.

(For large datasets, or papers with a very large number of statistical tests, you may upload a single table file with tests, Ns, etc., with reference to sections in the manuscript.)

Group allocation

- Indicate how samples were allocated into experimental groups (in the case of clinical studies, please specify allocation to treatment method); if randomization was used, please also state if restricted randomization was applied
- Indicate if masking was used during group allocation, data collection and/or data analysis

Please outline where this information can be found within the submission (e.g., sections or figure legends), or explain why this information doesn't apply to your submission:

COVID19 negative versus positive status was defined from the results of PCR test and/or antibody test. 'Sero-low' versus 'sero-high' groups were defined by seroconversion scores, with half of the COVID19 cohort being allocated to each group. This is explained in the Materials and Methods section.

Additional data files ("source data")

- We encourage you to upload relevant additional data files, such as numerical data that are represented as a graph in a figure, or as a summary table
- Where provided, these should be in the most useful format, and they can be uploaded as "Source data" files linked to a main figure or table
- Include model definition files including the full list of parameters used
- Include code used for data analysis (e.g., R, MatLab)
- Avoid stating that data files are "available upon request"

Please indicate the figures or tables for which source data files have been provided:

All data generated for this manuscript is made available through the online researcher gateway of the COVIDome Project, known as the COVIDome Explorer, which can be accessed at covidome.org. Differences between COVID19 negative and positive patients can be visualized in the 'Impact of COVID19' dashboards for each -omics dataset. Differences between sero-low and sero-high COVID19 patients can be visualized in the 'Impact of Seroconversion' dashboards. The mass spectrometry proteomics data have been deposited to the ProteomeXchange Consortium via the PRIDE partner repository with the dataset identifier PXD022817. The mass cytometry data has been deposited in Flow Repository under the link: https://flowrepository.org/id/RvFrSYioKeUdYHXdkTD9TQPAXt4PqdkB5eie82h11JgAGSCQIneLKpcKd81Nzgwq. The SOMAscan® Proteomics, MSD Cytokine Profiles, and Sample Metadata files have been deposited in Mendeley under entry doi:10.17632/2mc6rrc5j3.1. The RNA-seq data have been deposited in NCBI Gene Expression Omnibus, with a pending accession number to be available upon acceptance.
